# Direct pedicle screw fixation in infected vertebrae for acute pyogenic spondylitis: short-term outcomes from a single-arm retrospective study

**DOI:** 10.3389/fcimb.2026.1825345

**Published:** 2026-07-08

**Authors:** Yuxin Gao, Linan Wang, Xingyu Duan, Jiong Wang, Jiandang Shi, Zongqiang Yang, Peng Zhang, Hekun Liu, Zhiyun Shi, Ningkui Niu

**Affiliations:** 1 Department of Orthopedics, General Hospital of Ningxia Medical University, Yinchuan, China; 2 The First Clinical Medical College of Ningxia Medical University, Yinchuan, China; 3 Research Center for Prevention and Control of Bone and Joint Tuberculosis, General Hospital of Ningxia Medical University, Yinchuan, China; 4 Department of Pharmacy, General Hospital of Ningxia Medical University, Yinchuan, China; 5 Medical Experiment Center, General Hospital of Ningxia Medical University, Yinchuan, China; 6 Ningxia Key Laboratory of Clinical and Pathogenic Microbiology, General Hospital of Ningxia Medical University, Yinchuan, China

**Keywords:** anti-infective therapy, clinical efficacy, fixation and fusion of involved vertebrae, pyogenic spondylitis, surgical treatment

## Abstract

**Background:**

The safety and efficacy of placing pedicle screws within infected vertebral bodies during surgery for acute pyogenic spondylitis remains controversial. The conventional “skip-level” fixation strategy avoids instrumentation at the infected site but may compromise spinal biomechanical stability and alignment. Conversely, fixation that includes the infected vertebrae can provide immediate stability, facilitate deformity correction, and promote bone fusion, although it carries potential risks of infection recurrence and implant failure.

**Objective:**

To describe the short-term outcomes of direct pedicle screw fixation in infected vertebrae for acute pyogenic spondylitis, combined with radical debridement and targeted antimicrobial therapy.

**Methods:**

A retrospective analysis was conducted on 32 patients with pyogenic spondylitis who underwent surgical treatment between January 2021 and January 2025. All patients had a confirmed etiological diagnosis obtained via microbiological culture or next-generation sequencing (NGS) of surgical specimens and received individualized perioperative anti-infective therapy guided by clinical pharmacists. The surgical protocol included debridement of the infectious focus and posterior pedicle screw fixation incorporating the infected vertebrae, performed via single-stage posterior, single-stage combined anterior-posterior, or staged combined anterior-posterior approaches. Primary outcomes focused on perioperative adverse events, including implant-related complications, infection recurrence, and wound healing. Secondary outcomes assessed clinical efficacy and involved comparisons of Visual Analog Scale (VAS) pain scores, Oswestry Disability Index (ODI), inflammatory markers (erythrocyte sedimentation rate [ESR], C-reactive protein [CRP], and white blood cell count [WBC]), spinal Cobb angle, Frankel neurological grade, and bone fusion rates. Inflammatory markers and Cobb angle were assessed preoperatively and at 1, 3, and 6 months postoperatively; VAS and ODI were assessed preoperatively and at 1, 3, 6, and 12 months postoperatively; and bone fusion was assessed at 6 and 12 months postoperatively. Bone fusion was independently evaluated by two blinded assessors using Bridwell or modified Lenke criteria.

**Results:**

All 32 patients completed the 12-month follow-up. One osteoporotic patient developed asymptomatic minor screw loosening, and one diabetic patient experienced delayed wound healing that resolved after treatment. No implant failure, infection recurrence, or severe neurological complications occurred. Friedman tests demonstrated significant overall time effects for all dynamic indicators (all *P* < 0.0001): ESR (*χ²*(3) = 62.87, *W* = 0.65), CRP (*χ²*(3) = 67.22, *W* = 0.70), WBC (*χ²*(3) = 37.93, *W* = 0.40), Cobb angle (*χ²*(3) = 34.71, *W* = 0.36), VAS (*χ²*(4) = 95.90, *W* = 0.75), and ODI (*χ²*(4) = 127.02, *W* = 0.99). By 6 months, inflammatory markers had decreased markedly: ESR from 55.19 ± 31.05 to 6.00 (3.50, 12.25) mm/h; CRP from 34.10 (13.97, 85.15) to 2.12 (0.96, 3.90) mg/L; and WBC from 9.08 (5.75, 11.27) to 5.46 ± 1.01 ×10^9^/L. The Cobb angle improved from 7.94 (6.84, 14.22)° to 8.34 ± 2.43°. At 12 months, VAS improved from 4.00 (4.00, 5.00) to 1.00 (0.75, 1.00), and ODI decreased from 28.72 ± 8.82% to 5.00 (2.25, 8.00)%. Neurological function recovered to Frankel grade E in 93.8% (30/32) of patients. The overall descriptive fusion rate (successful fusion in at least one graft site) was 78.13% at 6 months and 93.75% at 12 months postoperatively.

**Conclusions:**

In this single-arm retrospective study of 32 patients with acute pyogenic spondylitis, direct pedicle screw fixation involving infected vertebrae was performed in conjunction with radical debridement and targeted antimicrobial therapy. At 12 months, patients demonstrated favorable short-term outcomes, including a low incidence of implant-related complications, significant improvements in pain and disability, a high radiographic fusion rate, and no observed infection recurrence. These findings provide preliminary evidence that this comprehensive protocol is a viable strategy for achieving favorable short-term outcomes in selected patients, and may serve as a practical reference for surgeons considering direct instrumentation in infected vertebrae.

## 1 Introduction

Pyogenic spondylitis (also referred to as pyogenic spondylodiscitis or vertebral osteomyelitis in the literature) is a destructive spinal infection caused by the direct invasion of pathogens into the vertebral bodies and intervertebral discs. The spectrum of causative microorganisms is predominantly non-specific, with Staphylococcus aureus being the most frequently identified. Other common pathogens include Escherichia coli, Streptococcus species, and Klebsiella species (Tali et al., 2015). Its annual incidence is relatively low, approximately 0.2 to 2.4 cases per 100,000 population. However, the disease often leads to severe pain, spinal instability, neurological impairment, systemic sepsis, and even mortality (Babic and Simpfendorfer, 2017; Henry et al., 2025). The cornerstone of treatment is timely and effective antimicrobial therapy. Nevertheless, surgical intervention becomes indispensable for patients presenting with structural compromise, progressive neurological deficits, or significant deformity (Baleriaux and Neugroschl, 2004; Guerado and Cervan, 2012).

The objectives of surgery are radical debridement to control the infection and the reconstruction of immediate and long-term spinal stability. Drawing from the experience in treating chronic specific infections like spinal tuberculosis, a widely accepted guideline strategy involves performing thorough debridement followed by pedicle screw fixation incorporating the involved, infected vertebrae, all under the coverage of standardized anti-tuberculous therapy (Jutte and Van Loenhout-Rooyackers, 2006; Wang et al., 2007; Jin et al., 2014). However, the direct translation of this concept to pyogenic spondylitis has sparked enduring controversy (Rutges et al., 2016; Di Rienzo et al., 2025). The root of this debate lies in the fundamental difference in their pathological processes. Tuberculosis is a relatively chronic, localized granulomatous inflammation, whereas pyogenic infection is an acute, diffuse suppurative process. The intense inflammatory microenvironment in pyogenic spondylitis theoretically poses a significantly higher risk for bacterial colonization and biofilm formation on the implanted pedicle screws, potentially leading to persistent or recurrent infection (Rodriguez-Merchan et al., 2021; Liu et al., 2023; Zacher et al., 2024). Consequently, the traditional and cautious approach often advocates for “skipping the infected segment” fixation—avoiding instrumentation at the infected level and fixing only the adjacent healthy vertebrae above and below—with the aim of reducing implant-associated infection risk. This strategy, however, may come at the cost of compromised spinal biomechanical integrity and residual local deformity (Okada et al., 2009; Fushimi et al., 2012; Scheyerer et al., 2022; Park et al., 2023).

In recent years, the application of rapid pathogen diagnostic technologies, particularly NGS, has significantly enhanced the sensitivity and timeliness of pathogen detection. This advancement has made perioperative targeted, pathogen-specific anti-infective therapy a viable option (van Dijk et al., 2014). Against this backdrop, a crucial scientific and clinical question arises: With the safeguard of intensified anti-infective therapy based on definitive etiological evidence, can the infected vertebrae in pyogenic spondylitis be safely incorporated into the fixation construct? This approach aims to achieve optimal spinal stability reconstruction and alignment correction while ensuring infection eradication.

To address this question, we retrospectively analyzed 32 patients with acute pyogenic spondylitis and definitive pathogen identification who underwent direct pedicle screw fixation involving infected vertebrae, combined with radical debridement and targeted antimicrobial therapy. The aim of this study was to describe the short-term clinical and radiological outcomes of this comprehensive treatment protocol, thereby providing observational data to inform the ongoing clinical debate regarding the role of direct instrumentation in infected vertebrae.

## 2 Materials and methods

### 2.1 General data

**Figure 1:**
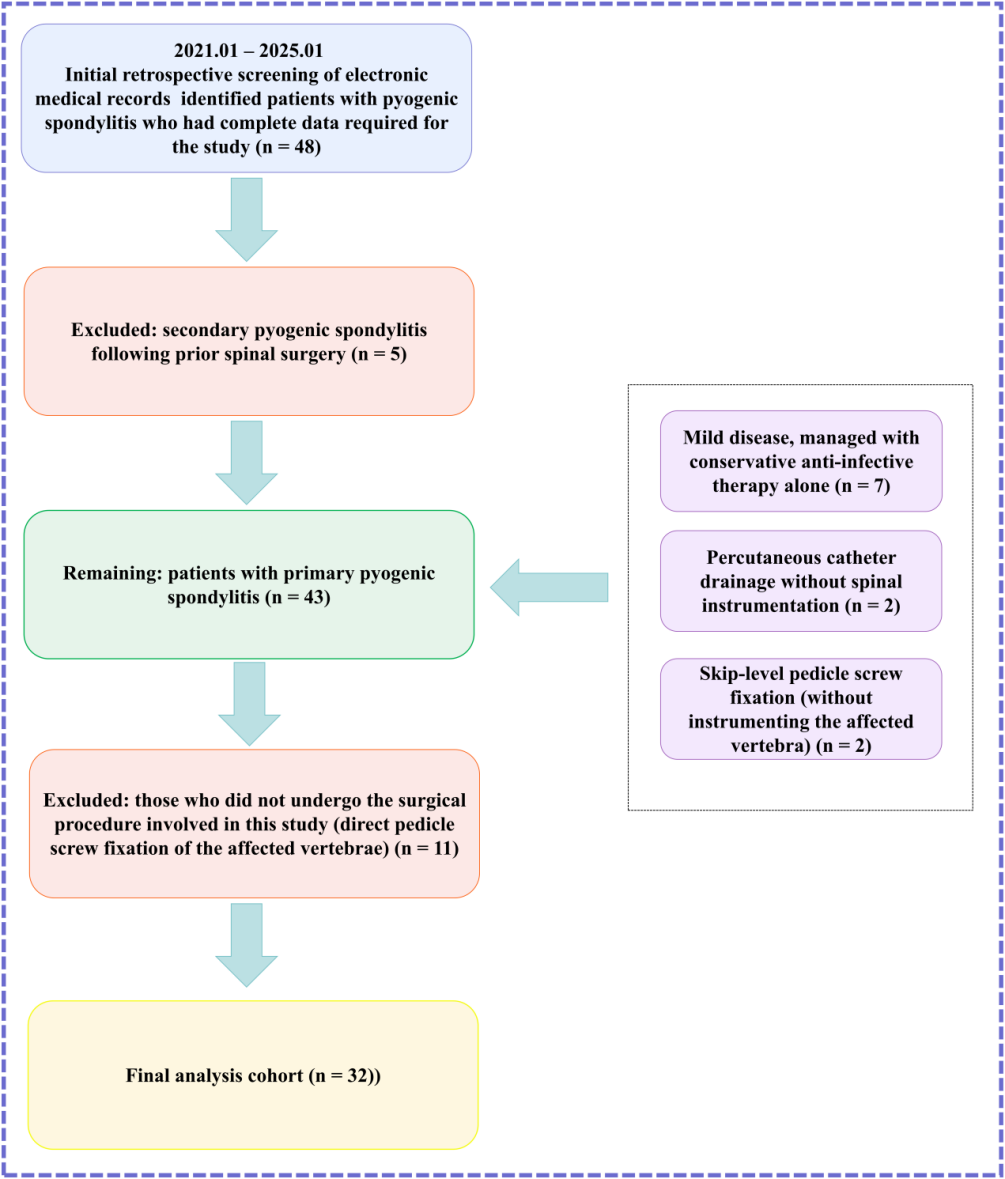
Patient selection flow diagram.

**Figure 2:**
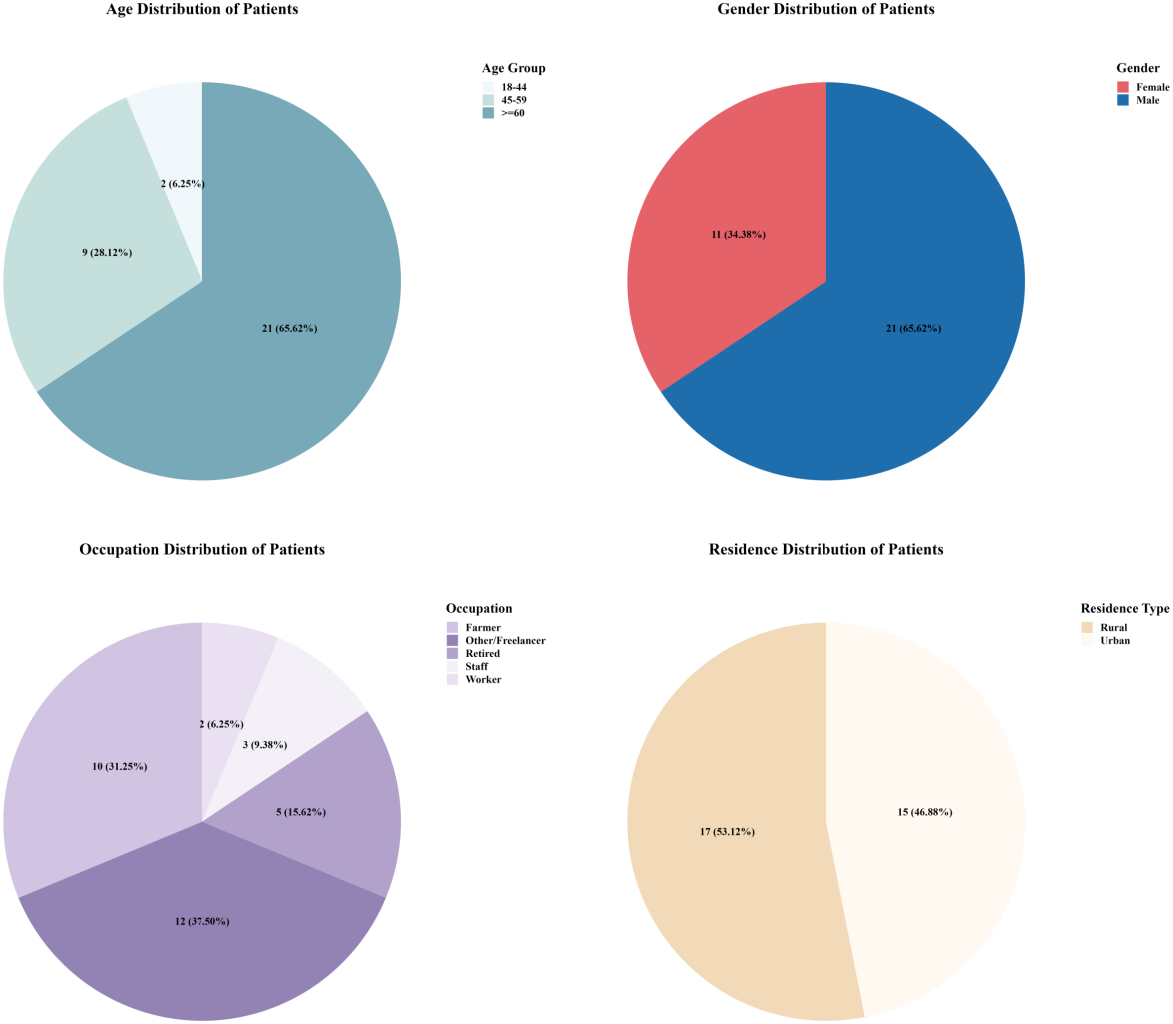
Distribution chart of patient demographic characteristics. Including patient age, gender, occupation, and place of residence.

**Figure 3:**
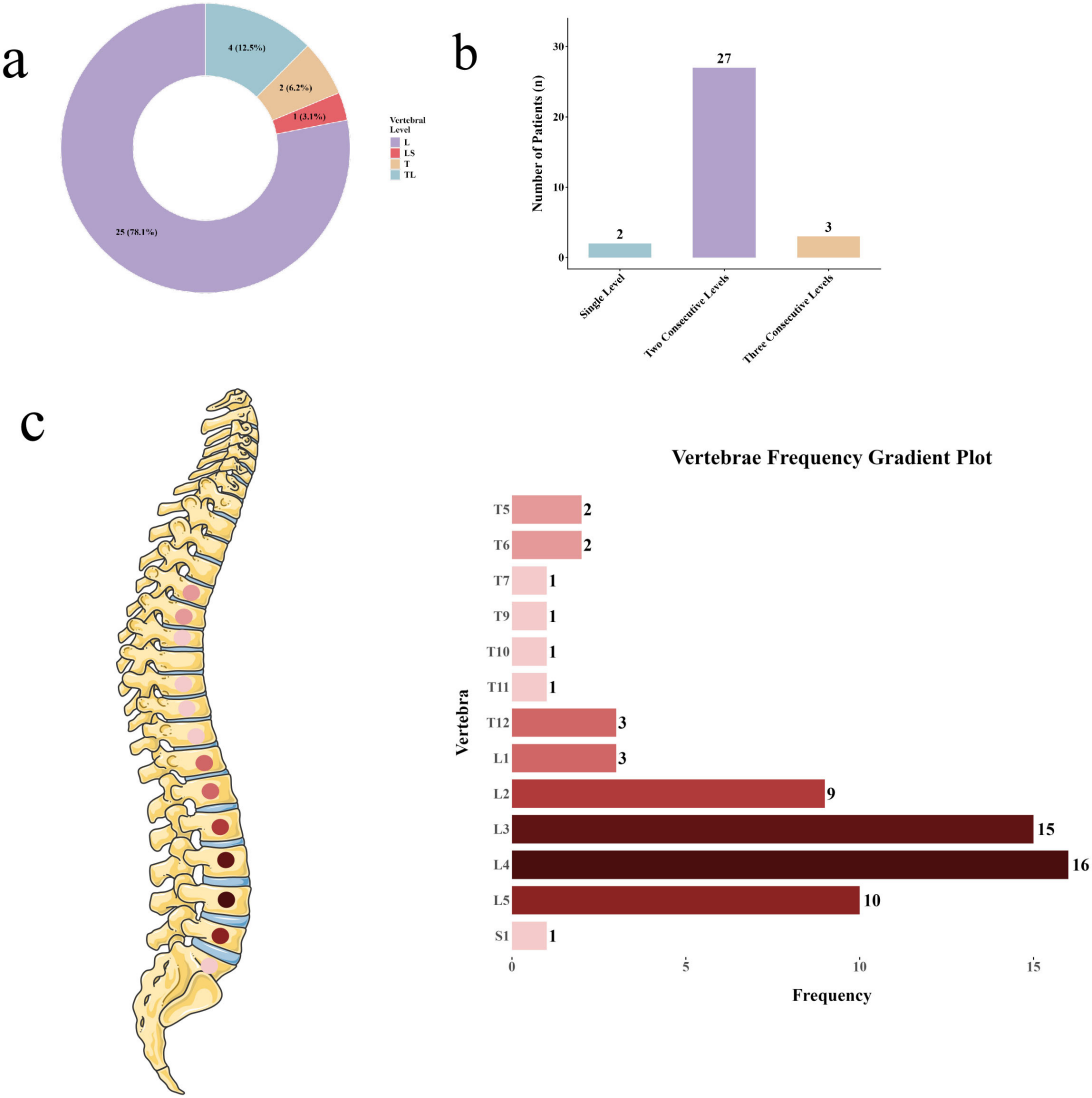
Visualization chart of lesion distribution in patients. **(a)** shows the spinal segments with the distribution of diseased vertebral bodies in patients; **(b)** shows the distribution of the number of diseased vertebral bodies; **(c)** shows the distribution of specific segments and quantities of diseased vertebral bodies. The darker the color, the higher the incidence rate in the corresponding region.

Through retrospective screening of the institutional electronic medical record system, 48 patients with a diagnosis of pyogenic spondylitis who were treated at our institution between January 2021 and January 2025 and possessed complete clinical documentation were initially identified. Among them, 5 patients with secondary pyogenic spondylitis resulting from prior spinal surgery were excluded. Of the remaining 43 patients with primary pyogenic spondylitis, 11 did not undergo the surgical procedure under investigation (direct pedicle screw fixation involving the infected vertebrae) and were excluded (**Figure 1**). The remaining 32 patients underwent the specified surgical procedure and had complete 12-month postoperative follow-up data available within the institutional electronic record system. Of these 32 patients, 21 were aged >60 years, 9 were aged 45–59 years, and 2 were aged 18–44 years. There were 21 males (65.62%) and 11 females (34.38%). A definitive diagnosis based on direct evidence from pathogenic microbiological culture or NGS was obtained for all included patients. The demographic information of the patients (including age, gender, occupation, and place of residence) is presented in **Figure 2**. Baseline patient characteristics (including etiology, medical history, clinical symptoms, etc.) are shown in **Table 1**. Laboratory and imaging examination features are summarized in **Table 2**. The distribution of involved segments and vertebrae is illustrated in **Figure 3**. Surgical-related data of all patients are summarized in **Table 3**. This study is a single-arm retrospective observational study. It was reviewed and approved by the Hospital Ethics Review Committee (Ethics Approval Number: KYLL-2025-1177). As a retrospective study, the requirement for informed consent was waived by the Ethics Committee.

**Table 1:** Baseline data of patients at admission.

Item	Number (n)	Percentage (%)
Diagnostic Methods		
- Bacterial Culture of Lesion Tissue	19	59.38
- Next-Generation Sequencing (NGS) of Lesion Tissue	13	40.63
Pathogenic microorganisms		
*- Coagulase-Negative Staphylococcus* (CNS)	4	12.50
*-Methicillin-Sensitive Staphylococcus aureus* (MSSA)	12	37.50
*- Methicillin-Resistant Staphylococcus aureus* (MRSA)	1	3.13
*- Streptococcus pneumoniae*	3	9.38
*- Streptococcus pyogenes*	1	3.13
*- Enterococcus faecium*	1	3.13
*- Escherichia coli*	6	18.75
*- Klebsiella pneumoniae*	3	9.38
*- Chryseobacterium gleum*	1	3.13
Past medical history		
- Hypertension	7	21.88
- Diabetes Mellitus (DM)	4	12.50
- Hepatitis History	2	6.25
- Long-Term Smoking History	4	12.50
- Long-Term Drinking History	2	6.25
Clinical features		
Fever Characteristics		
- High Fever	14	43.75
- Intermittent Fever	25	78.13
Spinal pain		
- Continuous Pain	7	21.88
- Intermittent Pain	25	78.13
- Hypoesthesia	18	56.25
- Radicular Pain	19	59.38
- Motor Dysfunction	21	65.63

Patients may present with multiple concurrent symptoms (e.g., high fever and intermittent fever, or hypoesthesia and radicular pain); therefore, percentages for clinical features do not sum to 100%.

**Table 2:** Laboratory examination results and imaging features of patients.

Item	Number (n)	Percentage (%)
Laboratory examination results		
- Elevated WBC	16	50.00
- Elevated ESR	24	75.00
- Elevated CRP	29	90.63
Imaging features		
- Spinal Deformity	10	31.25
- Kyphosis	1	3.13
- Scoliosis	9	28.13
- Paraspinal Abscess	15	46.88
- Unilateral	10	31.25
- Bilateral	5	15.63
- Intraspinal Abscess	10	31.25

**Table 3:** Perioperative operative parameters of patients.

Item	Number (n)	Percentage (%)
Surgical ethods		
- One-stage posterior fixation + posterior debridement	30	93.75
- One-stage posterior fixation + anterior debridement	1	3.13
- One-stage posterior fixation + two-stage anterior debridement	1	3.13
Bone graft fusion methods		
Intervertebral fusion materials		
- Autologous lamina bone + autologous cancellous bone + artificial bone	28	87.50
- Interbody fusion cage + autologous cancellous bone	2	6.25
- Autologous iliac bone	2	6.25
Posterior column fusion materials		
- Autologous cancellous bone + allogeneic bone	22	68.75
- Autologous cancellous bone + artificial bone	10	31.25
Operation Duration (min) (Mean ± SD)	163.66 ± 44.71	
Intraoperative Blood Loss (ml) (Mean ± SD)	356.06 ± 102.89	
Total Number of Fixed Vertebrae	93	
Total Number of Pedicle Screws	186	
Specific screw placement segments		
- T4	1	1.08
- T5	2	2.15
- T6	2	2.15
- T7	1	1.08
- T8	1	1.08
- T9	1	1.08
- T10	1	1.08
- T11	3	3.23
- T12	4	4.30
- L1	7	7.53
- L2	13	13.98
- L3	20	21.51
- L4	21	22.58
- L5	12	12.90
- S1	4	4.30

#### 2.1.1 Inclusion criteria

1. All patients had etiological evidence confirmed by pathogenic microbiological culture or NGS of specimens (tissue or pus) obtained via puncture or surgery, to ensure a definitive diagnosis of pyogenic spondylitis and exclude non-pyogenic specific spinal infections.
2. All patients underwent pedicle screw fixation that incorporated the infected vertebral body/ies, regardless of whether the procedure was an anterior debridement combined with posterior fixation or a posterior-only debridement, decompression, and fixation surgery.
3. All patients had complete medical records.

#### 2.1.2 Exclusion criteria

1. Patients without confirmed pathogenic microbiological or NGS evidence, i.e., those with unverified non-specific bacterial types, were excluded even if clinically diagnosed with pyogenic spondylitis. Patients with confirmed specific spinal infections, including spinal tuberculosis, Brucella spondylitis, and viral or fungal spondylitis, were also excluded.
2. Patients with secondary pyogenic spondylitis resulting from invasive procedures, including surgeries related to spinal degeneration.
3. Patients with incomplete data or those who refused to participate in the study.

### 2.2 Preoperative preparation and surgical decision algorithm

Preoperative anti-infective therapy was administered until systemic infectious symptoms had resolved, with regimens formulated under the guidance of clinical pharmacists. Perioperative supportive care included nutritional optimization and correction of comorbidities to ensure surgical tolerance.

The surgical strategy was determined according to a standardized algorithm based on three domains: ① spinal stability status and deformity severity, ② the necessity of anterior column reconstruction, and ③ patient general condition and surgical tolerance.

Definition of Spinal Instability. Radiographic instability was defined by any of the following: ① vertebral body collapse with loss of >50% of anterior or middle column height on sagittal CT; ② segmental kyphosis >15° or progressive deformity on serial imaging; or ③ extensive destruction of the intervertebral disc and adjacent endplates with anticipated inability to achieve spontaneous fusion. Clinical indicators included intractable mechanical pain refractory to conservative therapy, or dynamic motion-induced pain on flexion-extension radiographs.

Indications for Anterior Column Reconstruction. Anterior reconstruction was considered necessary when: ① the infected vertebral body demonstrated >50% cancellous bone loss or a cavitary defect >1 cm on CT; ② the intervertebral disc space was completely destroyed with endplate apposition loss; or ③ a large paraspinal or epidural abscess extending anterior to the dural sac required anterior drainage. For patients with circumferential neural compression, posterior decompression was combined with anterior reconstruction as indicated.

Surgical Approach Selection. All patients underwent posterior pedicle screw fixation incorporating the infected vertebrae. The approach was selected as follows:

Single-stage posterior approach: For patients with preserved anterior column height (>50%), no need for structural interbody support, and satisfactory general condition.

Single-stage combined anterior-posterior approach: For patients meeting anterior reconstruction criteria with satisfactory tolerance for a single session. Posterior fixation was performed first, followed by anterior debridement and structural grafting.

Staged combined anterior-posterior approach: For patients meeting reconstruction criteria but with compromised general condition (e.g., active sepsis requiring hemodynamic support, severe cardiopulmonary comorbidity, or poor nutritional status). Initial posterior fixation and debridement were performed as damage-control surgery, followed by delayed anterior reconstruction (typically 7–14 days later) after optimization of general status and infection control.

### 2.3 Surgical technique

The decision to instrument infected vertebrae was made intraoperatively after visual inspection and manual probing of the pedicle tract. Screws were inserted into infected vertebrae when: ① the pedicle cortical boundary remained intact on visualization and fluoroscopy; ② the vertebral body retained sufficient subchondral and cortical bone to achieve screw purchase (typically ≥2 cortical threads engaged); and ③ the infected segment was biomechanically critical for construct stability (bridging without it would result in a long lever arm >3 motion segments or inadequate sagittal alignment). Complete intraoperative debridement and targeted postoperative antimicrobial therapy were considered prerequisites for instrumenting the infected segment. If probing revealed complete pedicle destruction, screws were redirected to adjacent healthy vertebrae (this did not occur in the present cohort).

Posterior Approach. Under general anesthesia, a standard posterior approach was performed. Pedicle screws were implanted into infected and adjacent vertebrae under fluoroscopic guidance. Laminectomy was performed as clinically indicated, followed by thorough debridement of purulent material, necrotic disc, and destroyed bone. The surgical field was copiously irrigated with normal saline. Autologous bone graft (often combined with synthetic bone graft) was placed in the posterolateral region for thoracic levels or in the interbody and posterolateral regions for lumbar levels. Pre-contoured titanium rods were installed and a drainage tube was placed.

Anterior Approach (when indicated). A lateral extrapleural approach was utilized for thoracic lesions and a retroperitoneal approach for lumbar lesions. Radical debridement of pathological bone, disc, and granulation tissue was performed. After irrigation, an autologous tricortical iliac crest bone graft was impacted into the intervertebral space. Fluoroscopy confirmed graft position and a drainage tube was placed.

**Figure 4:**
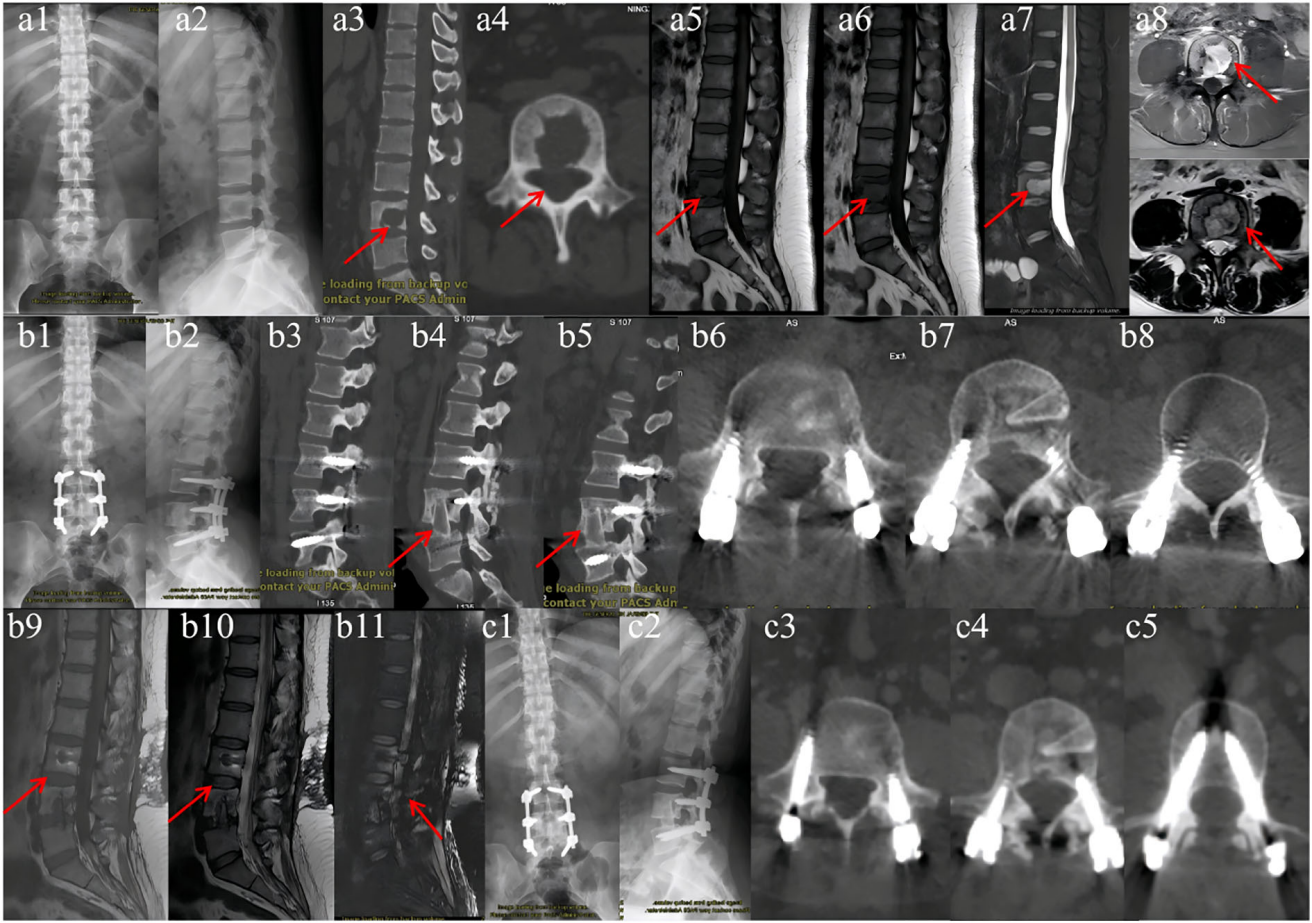
Preoperative and postoperative follow-up imaging of a patient with *coagulase-negative Staphylococcus* infection treated with direct pedicle screw fixation involving the infected vertebrae. Preoperative imaging: **(a1, a2)** Anteroposterior and lateral radiographs. **(a3, a4)** Sagittal CT images revealing bone destruction and a large cavity in the L4 vertebral body (arrows). **(a5–a8)** Sagittal and axial MRI demonstrating abscess formation in the L4 vertebral body and adjacent intervertebral space, with satisfactory vertebral body height (arrows). Postoperative imaging at 9 months: **(b1, b2)** Anteroposterior and lateral radiographs. **(b3–b8)** Sagittal and axial CT images showing good bone graft fusion at the site of previous bone destruction and cavity, with internal fixation in good position (arrows). **(b9–b11)** Sagittal MRI demonstrating no abscess recurrence (arrows). Postoperative imaging at 12 months: **(c1, c2)** Anteroposterior and lateral radiographs. **(c3–c5)** Sagittal CT images revealing good position of internal fixation and satisfactory bone graft fusion.

**Figure 5:**
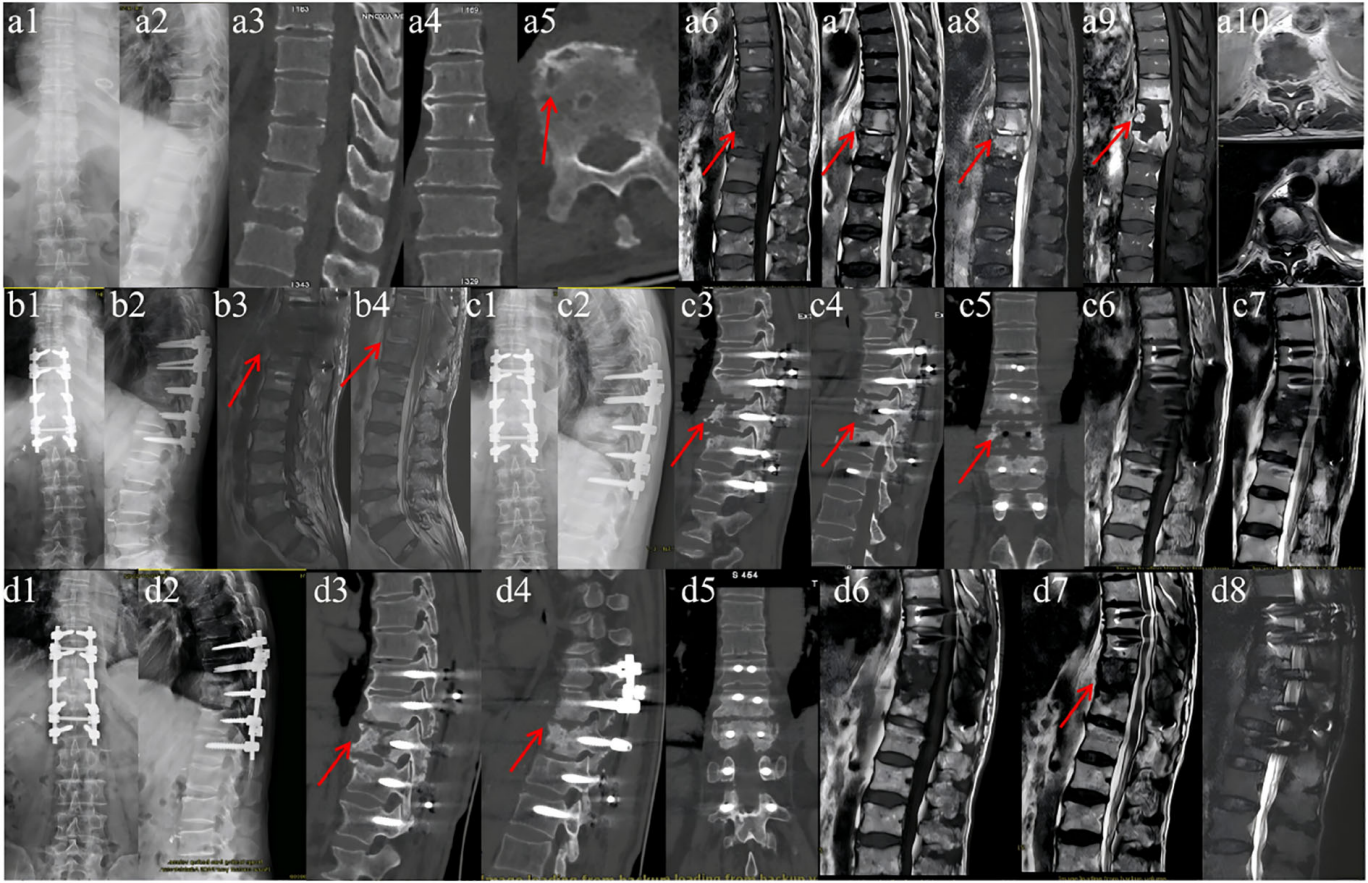
Preoperative and postoperative follow-up imaging of a patient with T9–T11 *Escherichia coli* infection treated with direct pedicle screw fixation involving the infected vertebrae. Preoperative imaging: **(a1, a2)** Anteroposterior and lateral radiographs. **(a3–a5)** Sagittal and axial CT images revealing micronodular bone destruction lesions (arrows). **(a6–a10)** Sagittal and axial MRI demonstrating bone marrow edema in the T9–T11 vertebral bodies, as well as abscesses in the intervertebral spaces and spinal canal, with severe destruction of the T10 vertebral body (arrows). Postoperative imaging at 1 month: **(b1, b2)** Anteroposterior and lateral radiographs. **(b3, b4)** Sagittal MRI indicating abscess clearance with no obvious recurrence (arrows). Postoperative imaging at 3 months: **(c1, c2)** Anteroposterior and lateral radiographs. **(c3–c5)** Sagittal and axial CT images revealing resolution of edema, the actual extent of bone destruction after abscess disappearance, and internal fixation in good position without loosening (arrows). **(c6, c7)** Sagittal MRI demonstrating no recurrence of abscess (arrows). Postoperative imaging at 12 months: (d1, d2) Anteroposterior and lateral radiographs. **(d3–d5)** Sagittal CT images demonstrating newly formed sclerotic bone in the lesion area, with internal fixation in good position and no fracture detected (arrows). **(d6–d8)** Sagittal MRI confirming that the patient achieved good recovery with no recurrence of abscess (arrows).

**Figure 6:**
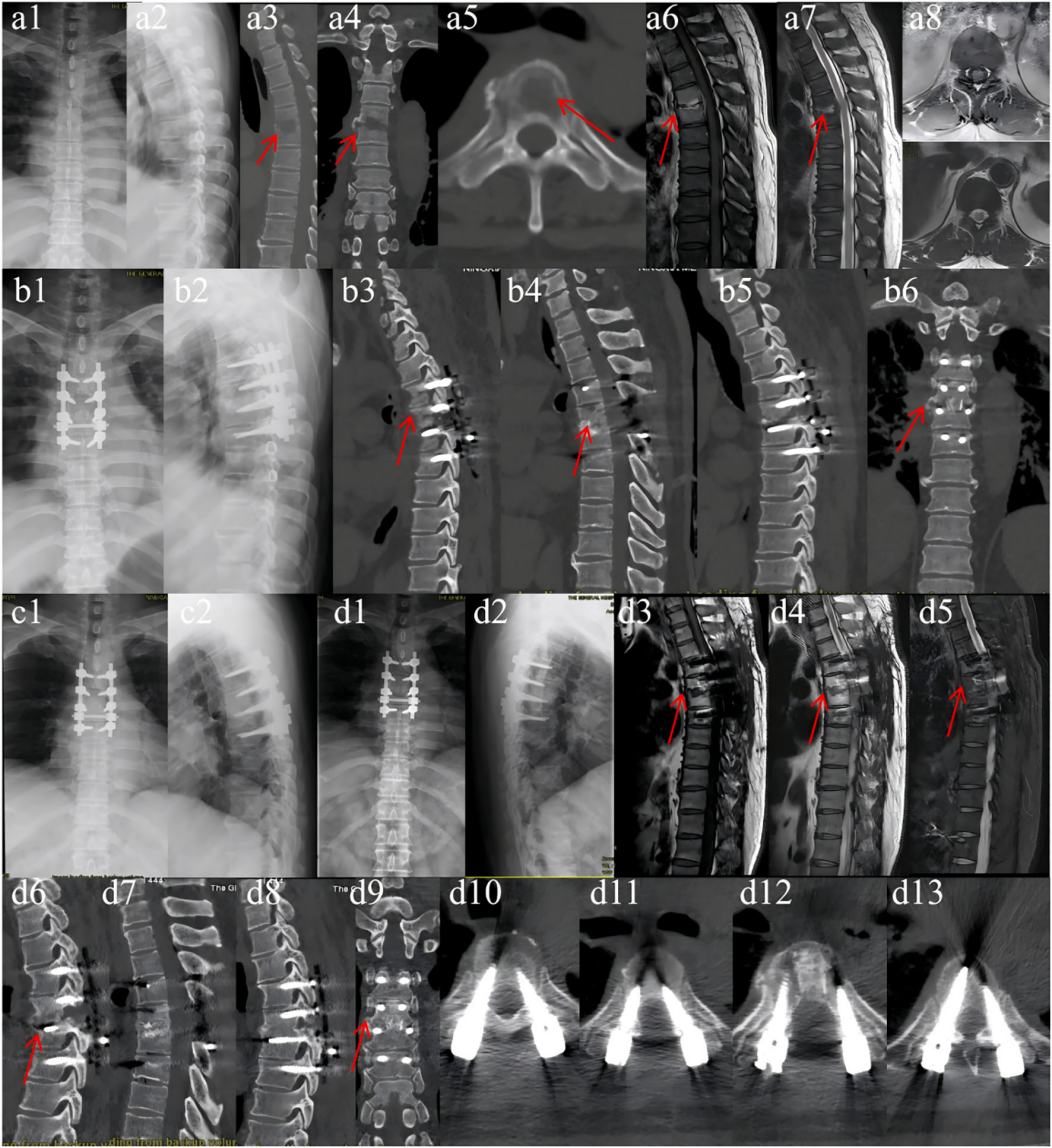
Preoperative and postoperative follow-up imaging of a patient with T5–T6 *Klebsiella pneumoniae* infection treated with direct pedicle screw fixation involving the infected vertebrae. Preoperative imaging: **(a1, a2)** Anteroposterior and lateral radiographs. **(a3–a5)** Sagittal and axial CT images revealing severe bone destruction at the T5–T6 vertebral interface and a cavity in the T6 vertebral body. **(a6–a8)** Sagittal and axial MRI demonstrating bone marrow edema in the T5–T6 vertebral bodies, along with intervertebral space and epidural abscesses. Postoperative imaging at 3 days: **(b1–b6)** X-ray and CT images. Postoperative imaging at 3 months: **(c1, c2)** Anteroposterior and lateral radiographs. Postoperative imaging at 9 months: **(d1, d2)** Anteroposterior and lateral radiographs. **(d3–d5)** Sagittal MRI demonstrating no recurrence of abscess. **(d6–d13)** Sagittal and axial CT images confirming that the internal fixation is in good position.

Representative preoperative and postoperative imaging studies from three illustrative cases are presented in **Figures 4**–**6**.

### 2.4 Postoperative management

Surgical specimens were sent for pathological, microbiological, and genetic sequencing analysis to guide antimicrobial therapy. Drainage tubes were removed when output was <50 mL for posterior approaches and <20 mL for anterior approaches. Incisions were dressed regularly; sutures were removed at 10–14 days, delayed for high-risk patients. Patients remained non-weight-bearing for 4–6 weeks. Ambulation with a brace was initiated only after radiographic confirmation of stable graft incorporation. The total duration of antimicrobial therapy was determined comprehensively based on clinical presentation, inflammatory markers, and imaging findings, with regimens optimized in consultation with clinical pharmacists. Follow-up assessments were conducted at 1, 3, and 6 months postoperatively, with subsequent visits every 6 months up to 12 months. All clinical and radiographic data were systematically archived for efficacy analysis.

### 2.5 Next-generation sequencing

Targeted next-generation sequencing (tNGS) was performed on surgical specimens (tissue or pus) when conventional microbiological culture yielded negative or inconclusive results. As our institution does not maintain in-house NGS capability, specimens were sent to a certified third-party clinical sequencing laboratory. The clinical team utilized the generated pathogen reports for therapeutic decision-making without direct involvement in wet-laboratory processing or bioinformatic pipeline operation. Following DNA extraction and quality control assessment at the referral laboratory, libraries were constructed and subjected to targeted enrichment using a hybridization capture-based protocol, followed by high-throughput sequencing on an Illumina platform. The bioinformatics pipeline included host sequence subtraction, low-quality read filtering, and alignment against a curated microbial reference database encompassing bacteria, fungi, and mycobacteria. Turnaround time from sample receipt to clinical report was typically 3–5 days.

To differentiate true pathogens from environmental contaminants or commensal flora, a multi-layered filtering strategy was applied: ① negative controls (no-template and extraction controls) were run in parallel to identify background contamination; ② species with extremely low read counts relative to the dominant pathogen and lacking clinical correlation were filtered out; ③ common skin commensals were interpreted cautiously and only considered significant when supported by concurrent culture or histopathological evidence. Polymicrobial infections were defined by the co-detection of multiple pathogenic species with comparable read abundance and clinical relevance.

Among the 13 patients who underwent tNGS, the results directly informed antimicrobial regimen adjustment in 10 cases (76.9%), including 7 cases where prior culture was negative and 3 cases where resistant or atypical organisms not covered by empiric therapy were identified. In the remaining 3 cases, tNGS confirmed the culture-identified pathogen, serving as corroborative evidence without necessitating regimen change.

### 2.6 Antimicrobial therapy protocol

The antimicrobial regimen was formulated and adjusted under the routine consultation of both clinical physicians specializing in spinal infection within our research team and clinical pharmacists. Preoperative intravenous (IV) antibiotics were administered until systemic infectious symptoms (e.g., fever and chills) had resolved. Postoperatively, targeted IV antibiotic therapy was continued for a mean duration of 5 weeks (range 4–6 weeks) after pathogen identification and susceptibility results were available. Transition from IV to oral sequential therapy was permitted when the following criteria were met: ① resolution of systemic infectious symptoms; ② declining trend of inflammatory markers (ESR and CRP) toward normal ranges; ③ no radiographic evidence of new or recurrent abscess; and ④ satisfactory wound healing without signs of local infection. The total duration of antimicrobial therapy was individualized based on clinical response and inflammatory marker normalization, with a mean total duration of approximately 12 weeks (range 8–16 weeks). Criteria for definitive discontinuation included: complete resolution of clinical symptoms, normalized or near-normal ESR and CRP, and radiographic confirmation of infection control without abscess recurrence.

### 2.7 Outcome measures and statistical analysis

The study outcomes were categorized into primary and secondary outcome measures.

Primary outcome measures focused on perioperative adverse events and infection control, encompassing: implant-related adverse events (e.g., loosening, breakage, or failure), imaging-confirmed abscess recurrence, clinical infection recurrence (manifested as fever and increased local pain), and abnormal wound healing (including non-healing, sinus tract formation, tissue necrosis, and purulent discharge).

Secondary outcome measures provided a multi-dimensional assessment of surgical efficacy and patient recovery. These included:

Perioperative data: surgical approach, fusion technique, graft material, operative time, intraoperative blood loss, and instrumented levels.

Clinical evaluation: Pain and functional status were assessed using the VAS and ODI preoperatively and at 1, 3, 6, and 12 months postoperatively.

Systemic inflammation: ESR, CRP, and WBC were measured preoperatively and at 1, 3, and 6 months postoperatively to monitor dynamic changes.

Radiographic assessment: The spinal Cobb angle was measured preoperatively and at each follow-up. Fusion assessment was performed at 6 and 12 months postoperatively. All 32 patients underwent both posterolateral fusion and interbody fusion. Posterolateral fusion was assessed bilaterally across the transverse processes and facet joints using the Modified Lenke Posterior Fusion Grades (**Table 4**) (Lenke et al., 1998; Lehr et al., 2022). Interbody fusion was assessed using the Bridwell Anterior Interbody Fusion Grades (**Table 5**), focusing on bony bridging between the graft and endplates, radiolucent lines, and graft remodeling (Bridwell et al., 1995; Bridwell et al., 2001). Given that all patients underwent both fusion procedures, fusion rates were analyzed and reported separately for each graft location. An overall descriptive fusion rate was calculated to indicate the proportion of patients achieving successful fusion in at least one graft site (posterolateral or interbody), but this rate should not be interpreted as a single homogeneous biological outcome. Neurological function: Preoperative and postoperative neurological status was evaluated using the Frankel grading system (**Table 6**) (Capaul et al., 1994).

**Table 4:** Modified Lenke posterior fusion grades.

Grade	Definition	Radiographic criteria
Grade A (Definite Fusion)	Bilateral solid fusion	Continuous trabecularized bone mass bridging bilateral transverse processes and facet joints; no radiolucent lines; mature bone density
Grade B (Probable Fusion)	Unilateral solid fusion with contralateral uncertainty	Thick unilateral fusion mass present; contralateral side partially visualized or indeterminate but no definite lucency; no hardware failure
Grade C (Possible Pseudarthrosis)	Incomplete or questionable fusion	Questionable lucent line or defect within fusion mass; incomplete bridging; graft resorption <50%
Grade D (Definite Pseudarthrosis)	Non-union	Graft resorption; hardware loosening/breakage; definite motion on dynamic X-rays

**Table 5:** Bridwell anterior interbody fusion grades.

Grade	Definition	Radiographic criteria
Grade I (Definite Fusion)	Solid arthrodesis with remodeling	Continuous trabecular bone bridging graft and adjacent vertebral endplates; graft incorporated with blurred margins; no radiolucent lines; no motion on dynamic X-rays
Grade II (Probable Fusion)	Possible solid arthrodesis	Graft appears incorporated but definite continuous trabecularization not visible; subtle/incomplete lucent lines (<2mm, non-progressive); graft outline visible but no collapse
Grade III (Possible Pseudarthrosis)	Lucency at graft-vertebra interface	Definite circumferential lucent area (“halo”) around graft; partial graft resorption or collapse; no definite trabecular bridging; minor motion on dynamic X-rays
Grade IV (Definite Pseudarthrosis)	Graft collapse with hardware failure	Significant graft collapse, displacement, or fracture; loosening/breakage of fixation; definite abnormal motion; progressive deformity or recurrent pain

**Table 6:** Scoring criteria for Frankel classification.

Grade	Grade description	Motor function	Sensory function
A	Complete Injury	Total loss of all motor functions below the injury level	Total loss of all sensory functions (pain, temperature, touch, etc.) below the injury level; no sensation in the saddle area (perineum)
B	Incomplete Injury (Sensation Only Preserved)	Total loss of motor functions below the injury level	Partial or complete preservation of sensory functions below the injury level, with the sensory preservation range exceeding the injury level; partial sensation may be preserved in the saddle area
C	Incomplete Injury (Partial Motor Function Preserved)	Partial motor functions exist below the injury level, but the muscle strength of key muscles (important muscles corresponding to nerve segments) is ≤ Grade 3 (unable to complete movements against gravity)	Partial or complete preservation of sensory functions below the injury level
D	Incomplete Injury (Good Motor Function)	Motor functions are preserved below the injury level, and the muscle strength of key muscles is ≥ Grade 4 (able to complete movements against gravity and certain resistance), but there are motor dysfunctions (e.g., decreased muscle strength, coordination abnormalities, sphincter dysfunction, etc.)	Partial or complete preservation of sensory functions below the injury level; sensory abnormalities (e.g., numbness, hyperalgesia, etc.) may exist
E	Normal Function	Motor function is completely normal, with no abnormalities in muscle strength, muscle tone, or coordination function	Sensory function is completely normal, with no sensory loss or abnormalities, and normal sphincter function

Statistical analysis was performed using R version 4.5.1 (R Core Team), GraphPad Prism 9.5.0, and Python (SciPy and statsmodels libraries). Normality of continuous variables at each time point was assessed using the Shapiro–Wilk test. All repeated-measures variables (ESR, CRP, WBC, Cobb angle, VAS, and ODI) exhibited marked positive skewness at one or more time points (characterized by SD > mean and/or Shapiro–Wilk P < 0.05). Consequently, the Friedman test was employed as the nonparametric alternative to repeated-measures ANOVA for all dynamic indicators.

*Post-hoc* pairwise comparisons between the preoperative and each postoperative time point were performed only when the overall time-effect test was significant. Dunn’s *post-hoc* tests with Bonferroni correction were applied (adjusted α = 0.05/k, where k is the number of *post-hoc* comparisons). Effect sizes were reported as Kendall’s *W* with 95% confidence intervals for all Friedman tests. Standardized mean differences (Cohen’s d) were calculated for significant *post-hoc* pairwise comparisons.

Inter-observer agreement for fusion grading was evaluated using Cohen’s kappa statistic. All statistical tests were two-sided. A significance level of α = 0.05 was used for overall time-effect tests; for *post-hoc* pairwise comparisons, the Bonferroni-adjusted significance level was applied.

## 3 Results

**Figure 7:**
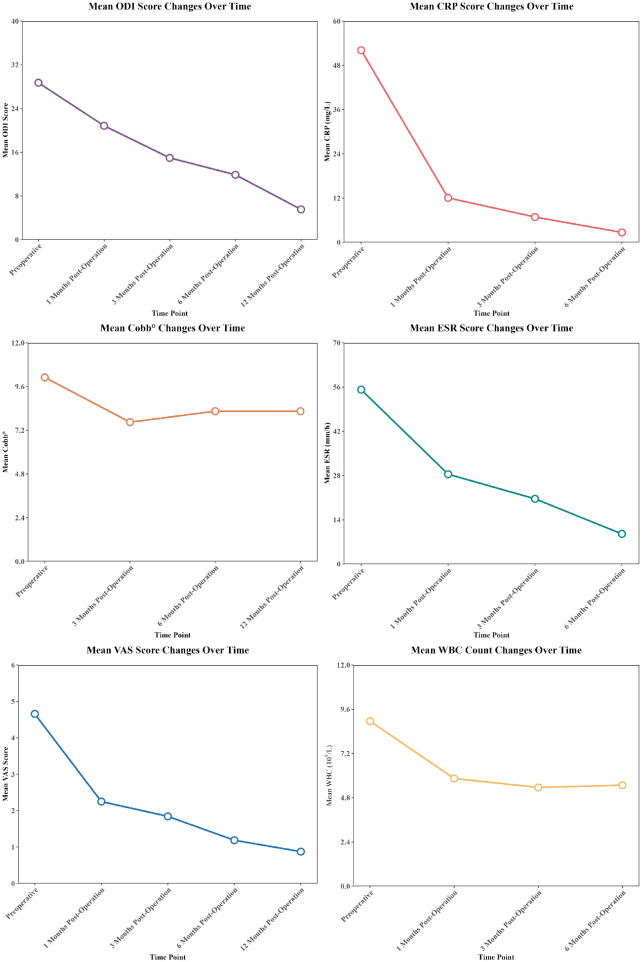
Line chart of changes in preoperative and postoperative efficacy indicators in patients. The horizontal axis represents various time points, and the vertical axis represents various observed efficacy indicators.

All 32 patients with pyogenic spondylitis who underwent surgical treatment completed the 12-month follow-up. The surgical outcomes were systematically evaluated according to the predefined primary and secondary outcome measures, with detailed data presented in **Tables 7**–**9** and **Figure 7**.

**Table 7:** Primary outcome measures in 32 patients.

Main outcome measure categories	Specific events	Number of occurrences(n=32)	Management and outcome
Internal Fixation-related Complications	Screw Loosening	1	Asymptomatic, under continuous follow-up
	Screw Fracture	0	—
	Overall Internal Fixation Failure	0	—
Infection-related Outcomes	Radiologically Confirmed Abscess Recurrence	0	—
	Clinical Infection Recurrence (fever, aggravated local severe pain)	0	—
Incision Healing Complications	Poor Incision Healing/Infection	1	Healed smoothly after debridement, dressing change, blood glucose control and smoking cessation
	Incision Sinus Tract Formation	0	—
Severe Neurological Complications	New-onset Paraplegia or Cauda Equina Syndrome	0	—

**Table 8:** Secondary outcome measures in 32 patients: preoperative and postoperative changes in observation indicators.

Time point	ESR (mm/h)	CRP (mg/L)	WBC (×10^9^/L)	Cobb angle (°)	VAS (points)	ODI (%)	Posterolateral fusion n (%)	Interbody fusion n (%)	Overall descriptive fusion rate n (%)
Preoperative	55.19 ± 31.05	34.10 (13.97, 85.15)	9.08 (5.75, 11.27)	7.94 (6.84, 14.22)	4.00 (4.00, 5.00)	28.72 ± 8.82	—	—	—
1 Month Postoperative	22.50 (7.50, 39.25)	8.82 (3.71, 16.58)	5.84 ± 1.35	7.00 (6.00, 9.00)	2.00 (1.75, 3.00)	20.84 ± 7.19	—	—	—
3 Months Postoperative	14.00 (3.00, 28.50)	2.58 (0.63, 5.92)	5.36 ± 1.60	8.25 ± 2.60	2.00 (1.00, 2.00)	14.97 ± 5.62	—	—	—
6 Months Postoperative	6.00 (3.50, 12.25)	2.12 (0.96, 3.90)	5.46 ± 1.01	8.34 ± 2.43	1.00 (1.00, 2.00)	11.00 (9.00, 15.00)	23 (71.88)	22 (68.75)	25 (78.13)
12 Months Postoperative	—	—	—	—	1.00 (0.75, 1.00)	5.00 (2.25, 8.00)	29 (90.63)	27 (84.38)	30 (93.75)
Test Statistic (df)	*χ²*(3) = 62.87	*χ²*(3) = 67.22	*χ²*(3) = 37.93	*χ²*(3) = 34.71	*χ²*(4) = 95.90	*χ²*(4) = 127.02	—	—	—
Effect Size (95% CI)	*W* = 0.65 (0.57–0.74)	*W* = 0.70 (0.62–0.78)	*W* = 0.40 (0.31–0.48)	*W* = 0.36 (0.28–0.44)	*W* = 0.75 (0.68–0.82)	*W* = 0.99 (0.92–1.00)	—	—	—
*P*-value	< 0.0001	< 0.0001	< 0.0001	< 0.0001	< 0.0001	< 0.0001	—	—	—

ESR, CRP, WBC, and Cobb angle were assessed at preoperative and 1, 3, 6 months; 12-month visit focused on VAS, ODI, and fusion. Overall Descriptive Fusion Rate only indicates that fusion has been achieved with one of the two bone grafting methods. It is solely used to describe the overall bone graft fusion rate and does not represent the specific fusion status of individual graft sites.

**Table 9:** Changes in patients’ neurological function.

Frankel grade	Preoperative (n)	12 months postoperative
A	0	0
B	0	0
C	2	0
D	14	2
E	16	30

Regarding the primary outcome measures, the incidence of implant-related adverse events during the follow-up period was low. Only one patient with osteoporosis and L3–4 level infection, who underwent L2–L5 pedicle screw fixation, was found to have minor loosening of the right L5 screw at the 12-month follow-up. This patient remained asymptomatic, with no associated local pain or neurological deficits, and continues under surveillance. No severe adverse events, such as implant breakage or catastrophic failure, occurred in any patient. Concerning infection recurrence, no patient exhibited radiographically confirmed recurrence of paravertebral or intraspinal abscess, nor any clinical signs of recurrence, such as fever or aggravated local pain. Only one wound healing complication was observed in a patient with a history of diabetes mellitus, who developed surgical site infection and delayed healing one month postoperatively. The incision healed successfully after standard wound care and debridement. Furthermore, no severe neurological complications attributable to disease progression, such as paralysis or cauda equina syndrome, were observed during the perioperative period or throughout the follow-up.

For the secondary outcome measures, laboratory inflammatory markers demonstrated rapid and sustained control of systemic inflammation. Friedman tests revealed significant overall time effects for all three inflammatory markers: ESR, *χ²*(3) = 62.87, *P* < 0.0001, Kendall’s *W* = 0.65 (95% CI: 0.57–0.74); CRP, *χ²*(3) = 67.22, *P* < 0.0001, Kendall’s *W* = 0.70 (95% CI: 0.62–0.78); and WBC, *χ²*(3) = 37.93, *P* < 0.0001, Kendall’s *W* = 0.40 (95% CI: 0.31–0.48). *Post-hoc* Dunn’s tests with Bonferroni correction showed significant reductions from preoperative values at 1, 3, and 6 months for all three markers (all adjusted *P* < 0.001), with large effect sizes (Cohen’s *d* = 0.86, 1.11, and 1.45 for ESR; 0.88, 0.90, and 1.04 for CRP; 0.82, 0.87, and 0.97 for WBC, respectively). ESR decreased from 55.19 ± 31.05 mm/h preoperatively to 6.00 (3.50, 12.25) mm/h at 6 months; CRP declined from 34.10 (13.97, 85.15) mg/L to 2.12 (0.96, 3.90) mg/L; and WBC normalized from 9.08 (5.75, 11.27) ×10^9^/L to 5.46 ± 1.01 ×10^9^/L.

Regarding pain and functional recovery, the Friedman test showed a significant overall time effect for VAS, *χ²*(4) = 95.90, *P* < 0.0001, Kendall’s *W* = 0.75 (95% CI: 0.68–0.82). *Post-hoc* Dunn’s tests with Bonferroni correction demonstrated significant reductions from baseline at 1, 3, 6, and 12 months (all adjusted *P* < 0.001), with large effect sizes (Cohen’s *d* = 2.75, 1.76, 2.49, and 2.56, respectively). VAS improved from 4.00 (4.00, 5.00) preoperatively to 1.00 (0.75, 1.00) at 12 months.

For ODI, the Friedman test showed a significant overall time effect, *χ²*(4) = 127.02, *P* < 0.0001, Kendall’s *W* = 0.99 (95% CI: 0.92–1.00). *Post-hoc* Dunn’s tests with Bonferroni correction demonstrated significant improvements from baseline at 1, 3, 6, and 12 months (all adjusted *P* < 0.001), with large effect sizes (Cohen’s *d* = 2.49, 3.13, 3.17, and 4.11, respectively). ODI decreased from 28.72 ± 8.82% preoperatively to 5.00 (2.25, 8.00)% at 12 months.

Radiographic structural assessment revealed that the Friedman test showed a significant overall time effect for Cobb angle, *χ²*(3) = 34.71, *P* < 0.0001, Kendall’s *W* = 0.36 (95% CI: 0.28–0.44). *Post-hoc* Dunn’s tests with Bonferroni correction showed significant improvement at 1 month (adjusted *P* < 0.001, Cohen’s *d* = 0.63), 3 months (adjusted *P* = 0.007, Cohen’s *d* = 0.62), and 6 months (adjusted *P* = 0.015, Cohen’s *d* = 0.56). Cobb angle improved from 7.94 (6.84, 14.22)° preoperatively to 7.00 (6.00, 9.00)°at 1 month, followed by a mild loss of correction to 8.25 ± 2.60°at 3 months and stabilization at 8.34 ± 2.43° at 6 months. This early loss of correction is a recognized phenomenon following spinal fusion surgery, attributable to graft settling and micromotion prior to solid arthrodesis. By 6 months, the Cobb angle had stabilized with a markedly reduced interquartile range, indicating a more consistent and stable sagittal alignment.

Neurological function assessment further validated the surgical efficacy. Among the 16 patients with neurological deficits preoperatively (2 Grade C and 14 Grade D), 14 had fully recovered to Frankel Grade E by the 12-month follow-up. To further characterize neurological recovery, the 16 patients with preoperative neurological deficits (Frankel grade C or D) were stratified by lesion location and presence of intraspinal abscess. Among them, 13 had lumbar lesions (L1–S1) and 3 had thoracic lesions (T4–T12). At 12 months postoperatively, 12 of 13 lumbar patients and 2 of 3 thoracic patients recovered to Frankel grade E. Regarding intraspinal abscess, 10 patients had preoperative abscess on MRI (**Table 2**); of the 8 within this subgroup who also presented with neurological deficits, 7 recovered to grade E. Among the remaining 8 patients with neurological deficits but no intraspinal abscess, 7 also recovered to grade E. Overall, 30 out of the 32 patients (93.8%) achieved Frankel Grade E, while only 2 patients (6.2%) remained at Grade D, demonstrating significant neurological recovery.Regarding bone fusion, all 32 patients underwent assessment of both posterolateral and interbody fusion at each follow-up time point. At 6 months, successful posterolateral fusion (modified Lenke grades A or B) was observed in 23 patients (71.88%), and successful interbody fusion (Bridwell grades I or II) was observed in 22 patients (68.75%). The overall descriptive fusion rate, indicating successful fusion in at least one graft site, was 78.13% (25/32). At 12 months, posterolateral fusion was achieved in 29 patients (90.63%), interbody fusion in 27 patients (84.38%), and the overall descriptive rate was 93.75% (30/32). CT confirmation of fusion was available in 24 patients (75.00%) at 6 months and 25 patients (78.13%) at 12 months; the remaining patients were assessed by dynamic radiographs and plain films. Inter-observer agreement for fusion assessment was evaluated using Cohen’s kappa on the initial independent assessments. For posterolateral fusion, kappa values were 0.86 (95% CI: 0.66–1.00) at 6 months and 0.84 (95% CI: 0.53–1.00) at 12 months. For interbody fusion, kappa values were 0.93 (95% CI: 0.79–1.00) at 6 months and 0.89 (95% CI: 0.68–1.00) at 12 months.

These results indicate that the surgical strategy effectively controlled the infectious process in patients with pyogenic spondylitis, with a low risk of implant-related adverse events, infection recurrence, and wound complications. Concurrently, it led to significant improvements in inflammatory markers, pain relief, spinal stability, neurological function, and daily activity capacity, demonstrating sustained and reliable therapeutic efficacy over the 12-month follow-up period.

## 4 Discussion

Pyogenic spondylitis, the most common non-specific spinal infection, is predominantly caused by non-specific bacteria. According to reported incidence rates, the most frequent pathogens are Staphylococcus aureus and Escherichia coli, followed by Streptococcus species and less virulent staphylococci (Skaf et al., 2010; Nui et al., 2022). The spectrum of pathogens identified in our study aligns with the reported epidemiology of pyogenic spondylitis (**Table 1**). *Staphylococcus aureus* was the most frequently isolated organism, accounting for 13 cases (40.63%) in total, comprising 12 methicillin-sensitive *S. aureus* (MSSA, 37.50%) and 1 *methicillin-resistant S. aureus* (MRSA, 3.13%). Other Gram-positive organisms included 4 *coagulase-negative Staphylococcus* (CNS, 12.50%), 4 *Streptococcus species* (12.50%) [3 *S. pneumoniae* (9.38%) and 1 *S. pyogenes* (3.13%)], and 1 *Enterococcus faecium* (3.13%). Gram-negative pathogens comprised 6 *Escherichia coli* (18.75%) and 3 *Klebsiella pneumoniae* (9.38%), along with 1 *Chryseobacterium gleum* (3.13%). Collectively, these 32 pathogen-confirmed cases demonstrated a bacteriological profile consistent with the established distribution of pyogenic spondylitis. This study included 32 patients, of whom 21 were male and 11 were female. Twenty-one patients (65.62%) were aged ≥60 years, 9 (28.12%) were between 45 and 59 years old, and 2 (6.25%) were <45 years old. These findings indicate a predominance of elderly male patients, which is consistent with the epidemiological characteristic of pyogenic spondylitis having a higher incidence in this demographic, as reported in the literature (Nui et al., 2022).

As a relatively uncommon infectious disease in clinical practice, conducting large-scale prospective clinical studies on pyogenic spondylitis is challenging. Consequently, current treatment strategies are often adapted from the experience managing chronic infectious diseases like spinal tuberculosis, and a widely accepted consensus based on high-level evidence is still lacking (Babic and Simpfendorfer, 2017). In terms of therapeutic principles, we fully endorse the established pharmacotherapy viewpoint: timely and effective anti-infective drug therapy forms the cornerstone of treatment, aimed at controlling inflammation early and preventing the spread of infection (Muckley et al., 2003; Butler et al., 2006; Skaf et al., 2010). The key lies in obtaining reliable etiological evidence to guide precise antimicrobial therapy. However, traditional microbiological methods, such as blood culture, often have limited sensitivity, which can lead to a degree of empiricism in initial treatment and potentially compromise efficacy (Baryeh et al., 2022; Astur et al., 2024). In recent years, the rapid development and application of molecular diagnostic technologies, particularly NGS, have significantly improved the sensitivity and timeliness of pathogen detection. This provides robust support for the early implementation of targeted anti-infective therapy, thereby enhancing the specificity and effectiveness of pharmacological treatment (Xu et al., 2022; Li et al., 2025; Tao et al., 2025). In this study, NGS was instrumental in identifying pathogens in 13 cases, effectively guiding the formulation of drug therapy regimens.

A study by T. Tsai et al. (2017) found that even in the absence of absolute surgical indications, early surgery can lead to better correction of kyphotic deformity and a shorter duration of antibiotic therapy. For patients presenting with progressive neurological impairment, severe spinal instability, significant kyphotic deformity, or failure of standard conservative treatment, surgical intervention becomes an indispensable key component. Its goals are to relieve neural compression, reconstruct spinal stability, correct deformity, and ultimately achieve infection control (Chen et al., 2007; Hamed et al., 2023). Open surgical approaches primarily include anterior, posterior, and combined anteroposterior routes, which can be performed in a single stage or in a staged manner. A.H. Fayazi et al. (2004) employed an anterior approach for debridement, followed by the placement of a titanium mesh cage for bone defect reconstruction; however, spinal stability was compromised until fusion was achieved. For patients with epidural or intervertebral abscesses accompanied by vertebral instability or neurological symptoms, posterior laminectomy combined with intervertebral debridement can achieve adequate decompression. However, the surgical disruption of the posterior elements may exacerbate spinal instability, necessitating supplemental posterior instrumentation to achieve long-term stability and facilitate bone fusion.

A long-standing controversy persists regarding whether pedicle screws should be placed directly into the infected vertebral bodies (the “involved vertebrae”) to achieve immediate mechanical stability. The traditional viewpoint holds that implants may serve as a substrate for bacterial biofilm formation, increasing the risk of persistent or recurrent infection (Zacher et al., 2024). In previous studies, many researchers, including J.J. Lee et al. (2022), predominantly employed a fixation strategy that “skipped” the infected segments. While effective for debridement, this method may not provide optimal stability in the early postoperative period, potentially limiting therapeutic outcomes. The biomechanical cost of the skip-fixation strategy may outweigh its theoretical infectious benefit. In a retrospective cohort study of lower thoracolumbar pyogenic spondylodiscitis, Nagata et al. (2020) found that skipping the most severely destroyed vertebra was associated with a significantly higher rate of pseudarthrosis revision surgery and required extension of the fixation range by approximately one vertebral level, yet failed to improve functional outcomes. Although avoiding screw placement in infected vertebrae may theoretically reduce the risk of biofilm formation, the consequent long-segment fixation and insufficient stability may instead increase the risk of early treatment failure. In all cases of the present study, we adopted a fixation strategy that placed screws in both the infected vertebrae and adjacent normal vertebrae, incorporating the diseased segments into a rigid fusion construct. This approach converts an unstable configuration with a long lever arm into a stable construct with a short lever arm, achieving immediate spinal stability and deformity correction while avoiding the biomechanical drawbacks associated with skip fixation. Recent advances in implant materials provide additional infectious considerations for direct instrumentation: titanium alloys inherently have a low propensity for bacterial adhesion, and surface modification technologies such as silver ion coating are being developed to inhibit initial bacterial colonization, promising to reduce implant-related infection risk without compromising osseointegration (Rodriguez-Merchan et al., 2021; Bohara and Suthakorn, 2022; Zacher et al., 2024). Furthermore, recent studies have shown that minimally invasive techniques such as UBE (unilateral biportal endoscopy) enable direct visualization and debridement of the lesion under continuous saline irrigation, achieving favorable short-term clinical outcomes and infection control in spinal infections. However, this technique requires a high level of operator proficiency, and its technical limitations must be considered when treating multilevel extensive bony destruction or when long-segment fixation is required (Chu et al., 2025; Ze et al., 2026). In this study, we performed open posterior fixation to achieve adequate debridement and multisegmental stability reconstruction. In all cases, definitive etiological diagnosis was obtained by microbial culture or NGS, based on which targeted anti-infective therapy was administered. Direct screw placement into the infected vertebrae was performed combined with intraoperative debridement. The clinical outcomes of this strategy were ultimately described using objective measures including postoperative inflammatory markers, fusion rates, and complications.

The results of this study demonstrated a rapid and significant reduction in pain, as evidenced by the VAS score decreasing from 4.00 (4.00, 5.00) preoperatively to 2.00 (1.75, 3.00) at 1 month postoperatively, and further to 1.00 (0.75, 1.00) at 12 months postoperatively. The Overall Descriptive Fusion Rate reached 78.13% at 6 months postoperatively and increased to 93.75% by 12 months, indicating that rigid internal fixation created favorable conditions for interbody fusion. On the premise that infection was effectively controlled through systematic perioperative anti-infective therapy, providing stable fixation for the infected segment facilitated pain relief, promoted osseous fusion, and aided neurological recovery. Regarding the primary outcome measures, only one patient with diabetes who continued smoking postoperatively experienced poor wound healing with purulent discharge. This condition improved rapidly after debridement, antibiotic adjustment, glycemic control, and smoking cessation. Another patient with L3–4 infection who underwent L2–L5 fixation was found to have minor loosening of the right L5 screw at the 12-month follow-up, though without apparent clinical symptoms, and remains under ongoing surveillance. Reduced bone mineral density in this patient may be a contributing factor to the screw loosening. No other implant-related complications were observed in the remaining patients. We posit that the establishment of mechanical stability may improve local blood supply, enhance host defense mechanisms, and increase antibiotic penetration at the site, potentially exerting a positive effect on infection control. Postoperative inflammatory markers, including WBC, CRP, and ESR, declined rapidly and returned to essentially normal levels by 6 months postoperatively. Furthermore, the significant improvement in the ODI and the maintained correction of the Cobb angle collectively substantiate the effectiveness of this comprehensive treatment strategy. Regarding neurological recovery, 14 of the 16 patients with preoperative deficits (2 Frankel Grade C and 14 Grade D) recovered to Grade E at 12 months, with 2 patients remaining at Grade D (**Table 9**). It is noteworthy that in our study, the incidence of spinal deformity in pyogenic spondylitis was relatively low with appropriate treatment. Only 9 patients presented with scoliotic deformity. The preoperative Cobb angle was 7.94° (6.84, 14.22) and was corrected intraoperatively. Pyogenic spondylitis typically manifests acutely with fever and severe back pain. In our cohort, 14 patients presented with high fever (>39 °C), 25 had intermittent fever, 7 suffered from severe persistent spinal pain, and 25 experienced intermittent spinal pain. The severity of early symptoms likely prompted earlier medical consultation, which may be one reason for the lower incidence and severity of spinal deformity observed. Additionally, studies suggest that pyogenic bacteria produce abundant digestive enzymes at the lesion site, activating the immune response, inducing inflammatory cell infiltration, and generating a large amount of inflammatory factors. This process may cause severe early destruction of soft tissues like intervertebral discs, while bony destruction of vertebral bodies is not as pronounced in the early stages, thus allowing for better preservation of spinal structural morphology (Gao et al., 2025).

Postoperative anti-infective therapy constitutes another critical component, where etiological confirmation is central to determining the treatment regimen. The application of sensitive antibiotics is significant for suppressing abscess recurrence and preventing implant-related infection (Zervou et al., 2015). The low recurrence rate observed in this study can be attributed not only to the thorough intraoperative debridement but also to the targeted antibiotic therapy based on definitive pathogen diagnosis. L. Bernard et al. (2015), in a large multicenter study, found that a 6-week course of antibiotic therapy was not inferior to a 12-week course, with the former offering better patient compliance and treatment adherence. We maintain that antibiotic therapy should adhere to individualized principles. The regimen employed in this study involved intravenous administration for at least two weeks preoperatively, followed by at least 4–6 weeks of intensive intravenous therapy postoperatively after pathogen confirmation, and subsequent transition to oral antibiotics as appropriate based on follow-up assessments. Efficacy and treatment duration were guided by regular laboratory and imaging evaluations to monitor for recurrence and ensure sustained therapeutic outcomes.

This study is a single-center, retrospective observational investigation and possesses certain limitations. Firstly, constrained by the very low incidence of pyogenic spondylitis, the sample size is relatively limited and lacks a randomized controlled design. All cases employed the strategy of instrumenting the involved vertebrae, without a control group utilizing a “skip-level” fixation or alternative surgical approaches for direct comparison. This limits the strength of the conclusions. Secondly, the study only included patients who successfully underwent surgery and had complete follow-up data, which may introduce survivorship bias. Consequently, the assessment of complication rates and therapeutic efficacy might be overly optimistic. Thirdly, this single-center retrospective study was limited to 32 patients, constraining statistical power and the ability to detect small effect sizes or perform robust subgroup analyses. The findings should therefore be interpreted as preliminary evidence, and validation through large-scale, multicenter prospective studies is warranted.

## 5 Conclusion

In this single-arm retrospective series of 32 patients with acute pyogenic spondylitis, direct pedicle screw fixation involving infected vertebrae was performed as part of a comprehensive treatment protocol encompassing radical debridement and targeted antimicrobial therapy. The 12-month follow-up data revealed a low incidence of perioperative adverse events, marked amelioration of pain and functional disability, satisfactory radiographic fusion in the majority of patients, and no documented instances of infection recurrence. Collectively, these observations offer preliminary evidence that, under the conditions of thorough surgical debridement and pathogen-directed antimicrobial coverage, incorporating infected vertebrae into the instrumented construct may constitute a viable surgical approach for carefully selected patients. These findings may inform preoperative counseling and surgical planning in similar clinical scenarios.

## Data Availability

The original contributions presented in the study are included in the article/supplementary material. Further inquiries can be directed to the corresponding authors.
